# Sulphate of Cinchonidia and Sulphate of Quinine

**Published:** 1877-06-20

**Authors:** Wm. Abram Love


### SOUTHERN MEDICAL RECORD.

VOL. VII. ATLANTA, GA., JUNE 20, 1877. NO. 6.

THOMAS S. POWELL, M.D., W. T. GOLDSMTIH, M. D., R. C. WORD, M. D., Editors.

R. C. WORD, Business Manager.

SUBSCRIPTION: TWO DOLLARS PER ANNUM, IN ADVANCE.

☞ All communications, and letters on business connected with THE RECORD for 1877 must be addressed to the Business Manager.

### Original and Selected Articles.

### SULPHATE OF CINCHONIDIA AND SULPHATE OF QUININE.

By PROF. WM. ABRAM LOVE, M. D.

To write an article on the subject of *sulphate of quinine* at this day and time, would seem to be a work of supererogation—especially in a section of country where that drug has so generally taken its place among ordinary household supplies—unless that article should be directed *against* its inordinate, indiscriminate, and injudicious use. In the hands of the profession, who have studied its physiological action, and the pathological conditions against which that action may be directed, it is one of the most valuable and potent remedies of the materia medica. But, unfortunately, from these very facts, it has passed into the hands of the laity who have not studied these things, and this most valuable remedy, from its abuse and injudicious use, has, in an indirect way, resulted in much evil to those who have, with too much faith, leaned upon it as a remedy in this mal-application. Yet the abuse of an article is no argument against its judicious use. This abuse, in certain sections of the country, has been in its administration as an anti-periodic and prophylactic, in controlling or preventing the development of miasmatic disease—in its use without the proper elimination from the system of such dead elements as have been disintegrated in the organism, while the emunctories have remained closed, preventing their exudation, or a failure of action, entire or in part, on the part of the glands and secreting membranes. Serious consequences follow this neglect, not from the use, but this abuse of the remedy—not that it fails of its legitimate action, but because too much is expected from its administration.

All is conceded that is claimed for it as a tonic, as an anti-periodic, as a supersedent, and a sedative (cardiac or otherwise), as well as much that is claimed for its prophylactic powers—yet we are told that it fails in many cases to come up to the expectations of those who give, or those who take it, or both. These failures may, as a general rule, be traced to the errors alluded to above, while in many cases they may be clearly attributable to error in the *time* of its administration. Particularly is this the case in intermittent, remittent and continued fevers. We had been instructed to administer the drug at stated intervals, immediately preceding the expected paroxysm in intermittent diseases—on the wane of the fever in remittents, and at stated periods, if at all, in continued forms of fever.

After all that has been claimed for quinine is conceded—there is another still, of which we hear but little—and that is its power of regulating the disturbance in the *normal* diurnal changes that take place in the system, under miasmatic and other paroxysmal diseases. The normal diurnal changes are two greater, and two lesser, with still four other, less marked, yet fixed as to time. They occur in health—are physiological. The two greater changes take place at midnight and at noonday. The two lesser at six o'clock morning and afternoon, and the other four, less marked, about midway between these four, but may predominate to the one or the other side according to the condition of the system, or the circumstances attendant upon each and every individual case. Now there is little hazarded in making the assertion that the potency of quinine depends—in all forms of disease diurnally paroxysmal, where it is at all admissible—upon its power of regulating the disturbances that take place in these diurnal changes, from the greatest to the smallest, and with equal confidence it may be stated that it is most effectively shown when the remedy is administered between the changes that take place at midnight, and the early or later morning change (i. e.) between one and ten o'clock a. m.

It is not intended to do more in this paper than to point to these changes, and to the fact of the more effective action of quinine when administered between the hours stated. If a patient will bear it at all with benefit, that benefit will be enhanced by taking advantage of this fact. Where the fever is continuous, and seemingly so high as to contra-indicate its use, the remedy will frequently, through its antipyretic action, reduce the temperature very much, and very certainly more than when given at any other period of the twenty-four hours. Any physician may, at any time, satisfy himself of this fact by a fair trial at the bedside, thermometer in hand, noting the diurnal thermal curves, and take advantage of the same in the treatment of any form of disease when the free use of quiniue is indicated. By resorting to this period for its administration, it will be found, too, that a far less quantity of the drug will be required to produce the same satisfactory results.

What is said here of quinine, may, with equal confidence, be said of the other alkaloid, or its salt, now presented to the profession—

THE SULPHATE OF CINCHONIDIA.

As to the difference in effect of the two remedies, administered in like cases, this will not be generally of a marked character. To some few points of difference it may not be out of place to refer, and in these there would seem to be some in favor of the cinchonidia. In the manifestations of its action upon the nervous system, they are less marked upon the nerves of special sense than those resulting from the use of quinine. There is less cerebral fullness, less ringing in the ears, less deafness and disturbance of vision, or vertigo.

In some individuals quinine has a tendency to produce muscular prostration, coldness of the surface, and in herculean doses, prostration and collapse. In this class of cases, in known individuals, where both have been tried, it has not been our province to mark any special difference between the action of the quinine and cinchonidia. As a cardiac sedative their effects are about the same. During a practice of very many years within the malarial belt, where miasmatic diseases in all their forms and complications presented themselves, from a simple chill and fever to the most aggravated form of hæmorrhagic malarial fever—(hæmaturia) and miasmatic purpurea hæmorrhagica, we found that when the disease was disposed to run into a chronic form, or a condition of *malarial cachexia*, quinine was not so effective in its action, not so certain or reliable as to results, as the preparations of bark containing other or all of the alkaloids, (i. e.) the solid extract, fluid extracts, tinctures, or even the powdered bark itself. In such cases the *cinchonidia*, it is believed, would be a more effective remedy than *quinine*.

Our work has been confined more particularly to the treatment of delicate females, many of them from the malarial belts of this and the adjoining States, who reach us with their systems surcharged with malaria, their vital forces depressed, their secretions deranged, their powers of assimilation and disintegration below par, their portal system passively engorged, and their nervous system very much impaired. In such cases we have found the cinchonidia, as we believe, more effective than the quinine, certainly more satisfactory to the patient from its affecting less the nerves of special sense, alluded to above. In such cases, where intermittents are disposed to assume a masqued form, cropping out under the guise of *neuralgia*, *myalgia*, or *rheumatism, etc.*—where a certain periodicity becomes observable in the train of nervous symptoms that complicate or accompany uterine diseases, the cinchonidia will present some advantages over the quinine—first, for the reasons assigned above, its tendency to produce less disturbance in the nerves of special sense; and for the additional reason that, while its action may be somewhat less rapid than quinine, it is, to the same degree, more persistent and permanent in its effects. Every practitioner who closely watches the effects of his remedies, has learned by experience that all alkaloids act more rapidly than the agents from which they are obtained. They act *explosively*, so to speak, there is a *tension* in their action that is quick but short-lived as compared with the agent from which they are derived. In the treatment of acute cases, and in cases where an *emergent* action is desired, this gives them—the alkaloids and their salts—great advantage over the original substance. But in a very large class of cases, where disease has assumed a chronic form, where it is persistent or recurrent, where there is a tendency to run into the condition of a *cachexia*, we will find the parent substance more effective than its more active alkaloid. We desire an action that is nearer allied to the slower process of combustion, rather than that of explosion. In such cases we resort to the original substance, to its solid extract, or to some form nearer approaching the original in its action, rather than the alkaloid or its salts. Now, as we approach the one or the other of these extremes in the form of the agent, we modify or intensify its action, but while its tension, its concentrated force, and, so to speak, explosive action, rests in the alkaloids and their salts, it loses, *pari-passu*, its persistence, and inasmuch as " strength does not consist in spasms, but in the stout bearing of burthens," we have occasion to recede a little from these more active preparations and their explosive, or, so to speak, spasmodic action, and trust to the stouter burthen-bearing powers of the preparations of nearer approach to the original mother agent.

Just at this point we feel that Messrs. Powers & Weightman, Manufacturing Chemists, have, as we stated before our State Medical Association last week, placed the profession and the people under lasting obligations to them, by furnishing an agent with which to fill one such gap—and a very important one—in our materia medica, in presenting to the profession sulphate of cinchonidia as a substitute for sulphate of quinine—an agent filling the gap just a degree below the tensive action of quinine, but at the same time more persistent in its ultimate effects.

For over twenty years of our professional life, it has been our province to battle against the effects of malaria in its most concentrated virulent form, within the malarial belts of this State, and we have constantly, during that period and since, felt the need of some stout burthen-bearing anti-periodical malarial antidote, without the explosive, tensive action of quinine. Though no longer of that malarial region, we bear a feeling recollection of its impresses, and while we are now in the higher altitude on the water-shed dividing the waters of the Atlantic and the Gulf streams (Atlanta is situated in 33° 45' 19.8" Lat., 84° 23' 29.7" Long.—at an elevation of 1,087 ft. above the level of the sea), where malaria is, comparatively speaking, scarcely known, yet from the nature of our professional labor, we have had ample opportunity to test the effects of the drug on cases (principally delicate females) coming from these malarial belts, or like regions of Georgia, Alabama, Florida, and South Carolina, where their systems had for months, and perhaps years, been constantly saturated with the subtle poison. In such cases we have found an advantage in the more persistent action of cinchonidia, in its less tendency to affect the nerves of the special senses, in the less sense of tension of the brain and nervous system, hence a better tolerance of its use—nor does it seem to produce the same amount of gastro-intestinal disturbance. In view of all these things, we can readily account for the fewer relapses that follow its judicious and sufficient use.

It is admitted that a change of air and change of altitude will, and does, aid in the restoration of those who come here from malarial regions for treatment of diseases and derangements peculiar to themselves or their sex, rather than their section. Yet they often reach us charged with malaria to such an extent that it would require long residence to eradicate the same without the aid of remedies to correct the existing morbid associations and mal-action in their organisms. Aside from their peculiar diseases or derangements, they bring with them the same pathological conditions, resulting from miasmatic influences, that existed with them at home, subject to the same laws, amenable to the same remedial agents. Such cases yield to the cinchonidia treatment in this respect, here, and a continuance of the same after their return home has, so far, given token of a like happy result, so that all the benefits in this respect cannot be attributable to the health-giving air of this Kennesaw range of mountains.

We have observed the same rules in regard to the use of cinchonidia that have so long governed us in the use of quinine. We have given it in the same doses, forms, and formulæ, and for the reasons given above, while in some few active and acute cases, where the active, tensive, explosive action of quinine would, to meet an emergency, or parry a paroxysm, seem to point to that as the preferable agent, still, in the vast majority of cases, where we are accustomed to regard quinine as indicated in the treatment, in which these exigencies do not exist, where the disease is chronic or the impress persistent, where the indications are for a seige treatment rather than by storm, we are persuaded that an advantage other than pecuniary will be found in the use of Sulphate of Cinchonidia.—*Atlanta Medical Journal.*

